# Divergent Effects of Mycobacterial Cell Wall Glycolipids on Maturation and Function of Human Monocyte-Derived Dendritic Cells

**DOI:** 10.1371/journal.pone.0042515

**Published:** 2012-08-03

**Authors:** Jolanta Mazurek, Lech Ignatowicz, Gunilla Kallenius, Stefan B. Svenson, Andrzej Pawlowski, Beston Hamasur

**Affiliations:** 1 Department of Microbiology, Tumor and Cell Biology, Karolinska Institutet, Stockholm, Sweden; 2 Department of Clinical Science and Education, Södersjukhuset, Karolinska Institutet, Stockholm, Sweden; Oklahoma Medical Research Foundation, United States of America

## Abstract

**Background:**

*Mycobacterium tuberculosis* (*Mtb*) is able to evade the immune defenses and may persist for years, decades and even lifelong in the infected host. *Mtb* cell wall components may contribute to such persistence by modulating several pivotal types of immune cells. Dendritic cells (DCs) are the most potent antigen-presenting cells and hence play a crucial role in the initial immune response to infections by connecting the innate with the adaptive immune system.

**Principal Findings:**

We investigated the effects of two of the major mycobacterial cell wall-associated types of glycolipids, mannose-capped lipoarabinomannan (ManLAM) and phosphatidylinositol mannosides (PIMs) purified from the *Mtb* strains H37Rv and *Mycobacterium bovis,* on the maturation and cytokine profiles of immature human monocyte-derived DCs. ManLAM from *Mtb* H37Rv stimulated the release of pro-inflammatory cytokines TNF, IL-12, and IL-6 and expression of co-stimulatory (CD80, CD86) and antigen-presenting molecules (MHC class II). ManLAM from *M. bovis* also induced TNF, IL-12 and IL-6 but at significantly lower levels. Importantly, while ManLAM was found to augment LPS-induced DC maturation and pro-inflammatory cytokine production, addition of PIMs from both *Mtb* H37Rv and *M. bovis* strongly reduced this stimulatory effect.

**Conclusions:**

These results indicate that the mycobacterial cell wall contains macromolecules of glycolipid nature which are able to induce strong and divergent effects on human DCs; *i.e* while ManLAM is immune-stimulatory, PIMs act as powerful inhibitors of DC cytokine responses. Thus PIMs may be important *Mtb*-associated virulence factors contributing to the pathogenesis of tuberculosis disease. These findings may also aid in the understanding of some earlier conflicting reports on the immunomodulatory effects exerted by different ManLAM preparations.

## Introduction

*Mycobacterium tuberculosis* (*Mtb*), the causative agent of human tuberculosis (TB), has co-evolved with the human host for millennia [1], [2]. Hence it is not surprising that mycobacteria have developed intricate mechanisms to interfere with the induction and course of the host immune response to the infection. At the earliest stage of infection mycobacteria-derived molecules, in particular those present in the outermost part of the cell envelope, interact with the cells of the innate immune response, macrophages (MΦ) and dendritic cells (DCs), and modify their cytokine production pattern, antigen presentation rate and response to T cell-mediated activation, including inhibition of different microbicidal mechanisms [3]. These early interactions of mycobacteria and mycobacteria-derived molecules with host cells are believed to shape and affect the subsequent adaptive immune responses. In particular, the cross-talk of *Mtb* with DCs is considered to be of profound consequence for the development of immunity as DCs, being the most potent antigen presenting cells, play a central role in triggering the adaptive immune responses against pathogens, including *Mtb.* In the natural *Mtb* infection the contact of bacteria with DCs typically first occurs on and in the mucosa of the airways and lungs. In the respiratory tract, DCs are distributed throughout the nasal mucosa to the lung alveoli. Here, the most prominent DC populations are localized within the epithelium of the conducting airways and within the lung parenchyma [4]. Following experimental intravenous infection of mice with *M. bovis* BCG, the bacteria infect splenic DCs and induce a transient IL-12 production and T cell stimulatory activity [5] and DCs are also infected by mycobacteria *in vitro* [6], [7].

Among mycobacterial immunomodulatory compounds, glycolipids of the bacterial cell wall, particularly lipoarabinomannans (LAMs) play a crucial role in modulating the hosts immune responses. LAMs are generally restricted to the mycobacterium genus and are found in the bacterial cell envelope of all mycobacterial species [8]. Generally they show a tripartite structure composed of an acylated glycosylphosphatidylinositol (GPI) anchor, attached to a poly-mannosyl backbone with arabinan branches, and different capping motifs [9]. The mannose-capped LAM (ManLAM), which is most abundant in slowly growing pathogenic species of the *Mtb* complex [8], [10], has been considered an important virulence factor contributing to the pathology of TB [9]. Other structural variants of LAM have been described: LAM with arabinan chains terminated with phosphatidyl-inositol motifs (PILAM) and LAM devoid of cappings (AraLAM), typical for less pathogenic mycobacteria e.g. *M. smegmatis* and *M. chelonae*, respectively [11]. PILAM has been reported to trigger production of IL-8, TNF and IL-12 and induces apoptosis, while AraLAM is not able to cause such responses [12], [13], [14].

In addition to LAMs, various other mycobacterial compounds have been shown to have potent modulatory effects *in vitro* on cells of the host immune system. These compounds include secreted or structural proteins such as ESAT-6 [15], 19-kDa lipoprotein [16], hsp65 and hsp70 [17] and cell wall associated glycolipids such as phenolic glycolipids [18], trehalose dimycolate (TDM), glycerol mono-mycolate [19] and the LAM precursors phosphatidyl-inositol mannosides (PIMs) 14,20,21,22,23 and lipomannan (LM) [14], [20], [24]. Some of these molecules, such as the 19-kDa lipoprotein, PIMs or LM, have been reported to bind to and signal through Toll-like receptors 2 and/or 4 (TLR2, TLR4) on host cells, while other, such as ManLAM are thought to bind DC-specific C-type lectin DC-SIGN [25] and mannose receptors and to deliver negative signals that interfere with TLR-mediated signaling [26].

The immunomodulatory activities of LAMs and other mycobacterial compounds have largely been proposed on the basis of the interaction of preparations from different mycobacteria and apparently of different degrees of purity, with isolated immune cells *in vitro*. It has become increasingly clear that certain *Mtb* complex strains spread more effectively and more often cause overt disease than others, and also induce different immune responses [27], [28]. Structural differences in mycobacterial glycolipids have been shown to account for differences in immune responses [22], [29], [30]. Hence, in order to dissect the role of mycobacteria derived molecules in innate immune responses the choice of mycobacterial strain for isolation of the glycolipids is of utmost importance.

Various ManLAM preparations have been reported to down-regulate T-cell proliferation [31], impede interferon (IFN)-γ-mediated activation of MΦ [32] affect classical complement activation [33], and scavenge bactericidal oxygen radicals [34]. In early studies ManLAM purified from bacteria of the *Mtb* complex have been shown to stimulate pro-inflammatory cytokines, in particular tumor necrosis factor (TNF), both in human [35], [36] and mouse [35], [37], [38], [39] MΦ.

The interaction of ManLAM alone or together with other mycobacterial glycolipids such as PIMs with DCs, in particular human DCs, is less well studied [25], [26], [40]. Here we analyzed the effects of highly purified ManLAM and PIMs isolated from *Mtb* H37Rv and *M. bovis* on the maturation of human DCs *in vitro* and their effects on LPS-induced cytokine production by human DCs.

## Results

### Isolation, Purification, Chemical and Immuno-chemical Characterization of ManLAM and PIMs from *Mtb* H37Rv and *M. bovis* Pasteur 38152

Several procedures have been described by different laboratories for the purification of LAM and other mycobacterial glycolipids [40], [41]. Generally, these procedures rely on the initial extraction of dried bacterial cells or cell walls with a lipophilic organic solvent/water system, followed by hydrophobic interaction and size exclusion chromatography in the presence of a detergent. These purification methods may result in complex glycolipid fractions of varying compositions which are not limited to mannosyl-terminated glycolipid species alone.

In our laboratory we routinely purify ManLAM using a rapid procedure in which a Concanavalin A-affinity column is employed in one of the steps to assure that only glycolipids with mannosyl-capped termini are isolated. Such glycolipids are further purified by hydrophobic interaction chromatography on Phenyl-Sepharose. On line SDS-PAGE analysis of glycolipid preparations from strains H37Rv, *M. bovis* Pasteur 38152 and *M. bovis* BCG (strain Copenhagen) revealed the presence of two bands upon silver nitrate staining; one band in the 20–30 kDa range, typical of ManLAM, and another band in the 6–10 kDa region (data not shown). The low molecular weight (LMW) band resisted digestion with proteinase K and disappeared upon treatment with alkali, which confirmed the non-protein, glycolipid nature of the compound(s) in this band. The ManLAM and the LMW glycolipid(s) were separated by an additional size exclusion chromatographic step in the presence of deoxycholate.

#### SDS-PAGE comparison of in-house ManLAM with TBVTRM reference ManLAM

**Figure 1:**
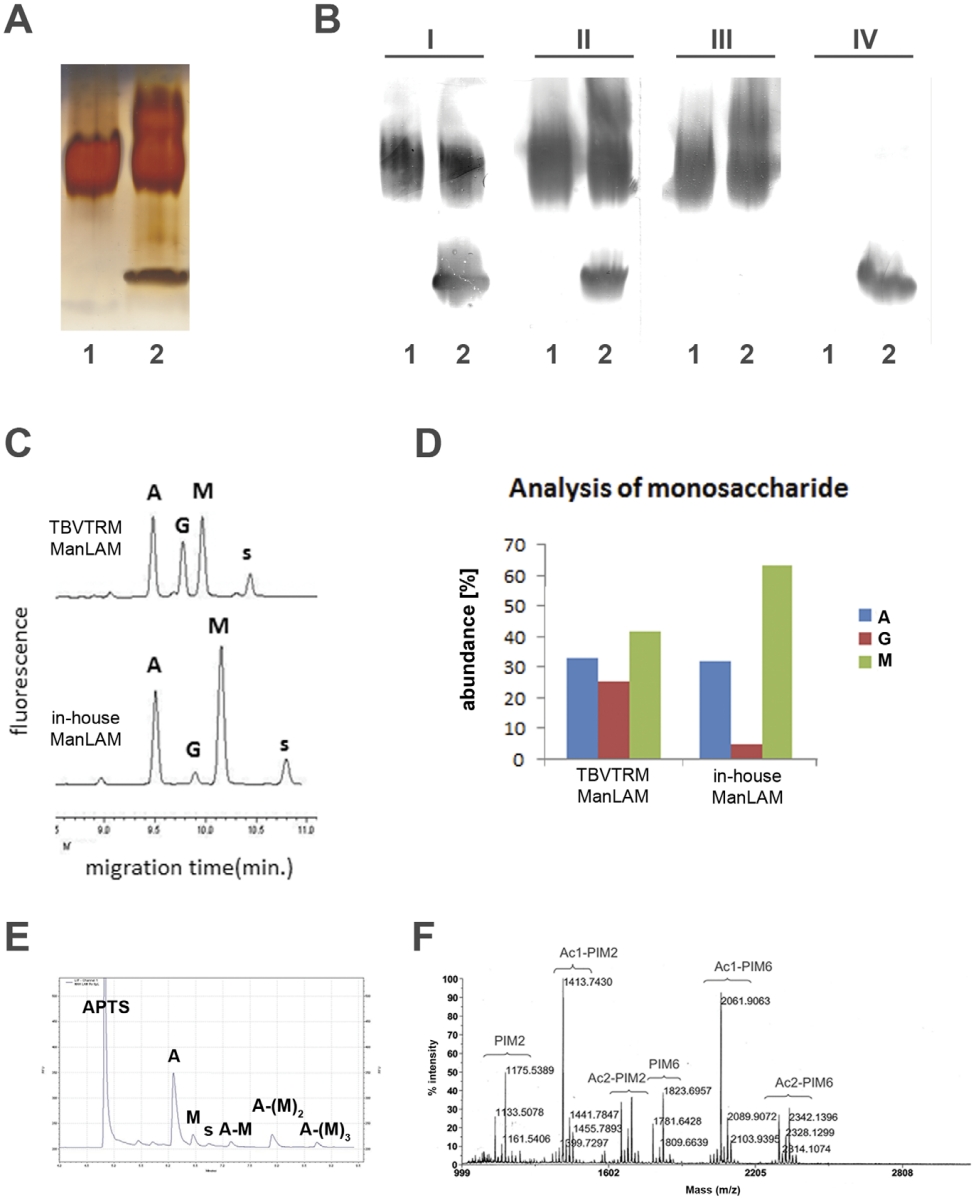
Characterization of ManLAM and PIM. SDS-PAGE gel stained with periodic acid-silver nitrate method (**A**) and Western blots (**B**) of in-house ManLAM (all lanes 1) and reference ManLAMs from TBVTRM Collection (panel B, I, lane 2: lot 08.Rv.1.24.ke1; panel A, lane 2 and panel B, II–IV, lanes 2: lot 09.Rv.2.9.8.ks). ManLAMs were detected using MAb KITB24. LMW glycolipid fraction from *Mtb* H37Rv cell wall was detected with MAb KITB51 that reacts with PIM. KITB24 and KITB51 were used either in combination (I and II) or separately: KITB24 alone (III), KITB51 alone (IV). (**C**) and (**D**) CE-LIF analysis of ManLAM-derived sugars. ManLAM was subjected to total acid hydrolysis and the resulting sugars were derivatized with APTS and subjected to capillary electrophoresis (**C**) and abundance of specific monosaccharides presented as % (**D**). A – arabinose, G – glucose, M – mannose, s – internal standard (heptose). (**E**) CE-LIF analysis of in-house ManLAM-derived capping mono-, di-, and trimannoside oligosaccharides, designated as as A-M, A-(M)_2_, and A-(M)_3_, respectively. A – arabinose, M –mannose, s - internal standard (heptose). (C–E courtesy of Dr. J. Nigou) (**F**) MALDI-TOF-MS analysis of LMW glycolipid fraction from our in-house *Mtb* H37Rv cell wall in negative-ion mode. This fraction designated PIM contained a mixture of PIM_2_ and PIM_6_ isoforms differing in number of fatty acyl substituents: PIM_2_ and PIM_6_; diacyl; Ac1-PIM_2_ and Ac1-PIM_6_; triacyl, Ac2-PIM_2_ and Ac2-PIM_6_; tetraacyl (courtesy of Drs. M. Gilleron and J. Nigou).

Our final in-house purified ManLAM preparations were analyzed by SDS-PAGE followed by periodic acid-silver nitrate staining and compared with a lot of the commonly used reference ManLAM preparations obtained from TB Vaccine Testing and Research Materials (TBVTRM) Collection (Colorado State University, Colorado, USA) (Figure 1A). While as expected the in-house ManLAM preparation only showed the typical 20–30 kDa range band (Figure1A; lane 1) the TBVTRM reference preparation (Figure 1A, lane 2) in addition showed at least two more bands indicating a heterogeneous preparation: one band of higher molecular weight than ManLAM and another in the 6–10 kDa region, characteristic for PIMs.

#### Immunochemical characterization

In immune dot blots monoclonal antibodies (MAbs) KITB24 (specific for ManLAM) and KITB51 (specific for PIMs) both reacted repeatedly with several reference ManLAM preparations from the TBVTRM Collection (data not shown). Next, Western blot analysis was performed using MAbs KITB24 and KITB51 in which our in-house preparations of ManLAM and PIMs were compared with two different batches of TBVTRM reference ManLAM preparations (Figure 1B). This analysis revealed that our in-house ManLAM preparation, as expected, only reacted with MAb KITB24 (Figure 1B), while the TBVTRM reference ManLAM preparations contained two bands reacting with MAbs KITB24 and KITB51, respectively, indicative of presence of both ManLAM and PIMs (Figure 1B).

#### Sugar analysis

Sugar analysis of in-house ManLAM and TBVTRM reference ManLAM (lot 08.Rv.1.24.ke1) preparations by capillary electrophoresis with laser-induced fluorescence (CE-LIF) detection after 8-aminopyrene-1,3,6-trisulfonic-acid (APTS) tagging (Figure 1C–E) after total acid hydrolysis revealed the expected sugars, mannose and arabinose, in both preparations. However, while our in-house preparation only contained trace amounts of glucose the TBVTRM preparation revealed considerable amounts of glucose (Figure 1C and D). The two preparations also showed different mannose/arabinose ratios. While almost equimolar in the TBVTRM preparation, the mannose/arabinose ratio was higher in our in-house ManLAM preparation indicative of a higher degree of mannose capping. The in-house ManLAM was further characterized by CE-LIF of APTS-tagged saccharides after mild acid hydrolysis. CE-LIF of ManLAM revealed characteristic proportion of monomannoside, dimannoside and trimannoside caps (Figure 1E).

#### Mass-spectrometric analysis

MALDI-TOF mass spectroscopic analysis of our in-house LMW glycolipid preparation showed the presence of the expected peaks typical of several acyl-isoforms of PIM_6_ and PIM_2_ (Figure 1F). This LMW glycolipid fraction purified from H37Rv or *M. bovis* Pasteur 38152 cell walls, respectively, is further in this paper referred to as PIMs. In all our subsequent studies on human DC responses PIMs were used without further separations into acyl-isoforms.

When the TBVTRM ManLAM preparation was passed through a HiTrap Phenyl-Sepharose column, we repeatedly found that about 50% of the material went through the column unretained (data not shown). This unretained fraction did not coat ELISA wells but was shown to maintain AM reactive epitopes in a sandwich ELISA, using MAb KITB24 as capture antibody and biotinylated MAb KITB29 as detecting antibody (data not shown).

### Effects of *Mtb* ManLAM and PIMs on Maturation of DCs

DCs derived from monocytes of healthy blood donors were cultured in the presence of *Mtb* H37Rv ManLAM or PIMs or/and LPS and the surface maturation markers levels were analyzed by flow cytometry. Pilot experiments revealed that the activation markers CD80, CD86 and MHC II molecules reached a plateau after 24–48 h of LPS stimulation (data not shown). All subsequent DC maturation experiments were performed after 48 h stimulation.

**Figure 2:**
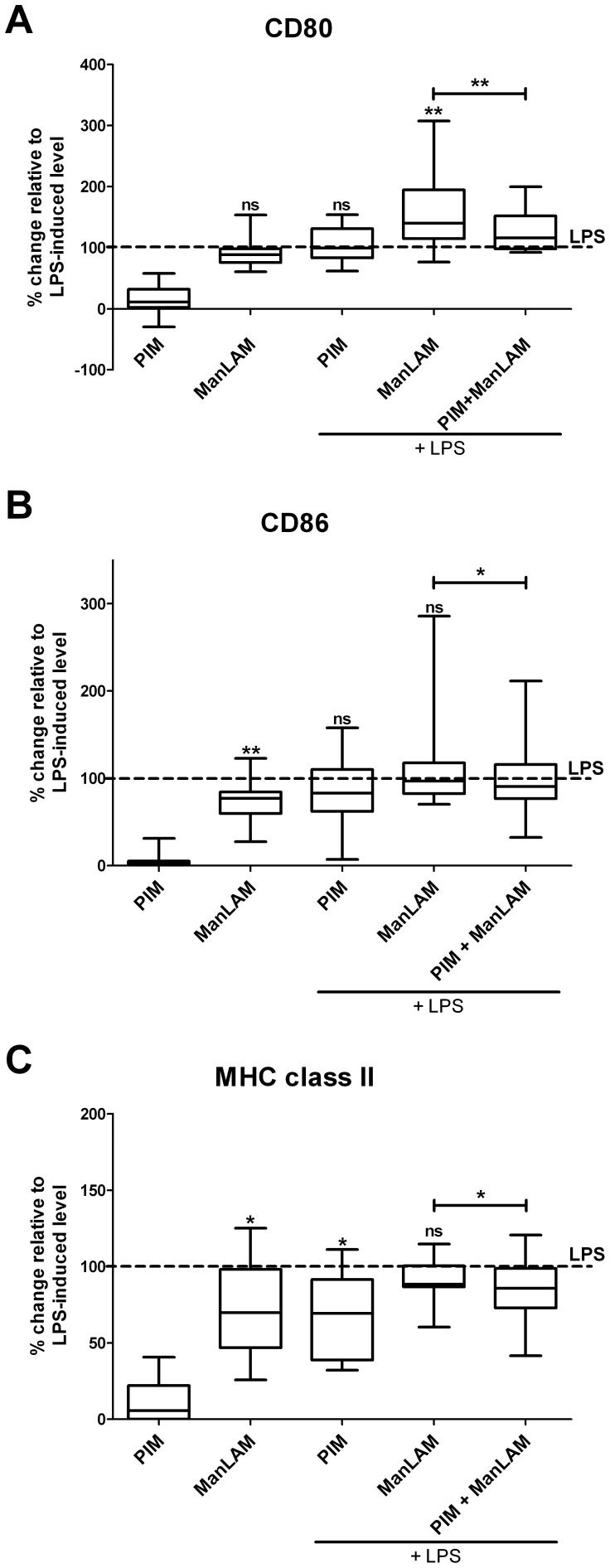
Maturation markers on DCs exposed to LPS and/or ManLAM and PIM from H37Rv. Immature DCs were stimulated with mycobacterial glycolipids (ManLAM and PIM at the concentration of 10 µg/ml and 5 µg/ml, respectively) or/and LPS (100 ng/ml) as indicated in the diagrams. After 48 h cells were harvested, stained for maturation markers CD80, CD86, and MHC class II and analyzed by flow cytometry. For each treatment mean fluorescence intensities (MFI) were related to the levels obtained for stimulation with LPS and expressed as %. The median percentage change in MFI is shown as a line. The box defines the 75th and 25th percentiles and the whiskers define the maximum and minimum values of 7–11 donors/group. The horizontal, dashed lines represent surface marker expression obtained for LPS treated DC. Groups significantly different from LPS-treated control are labeled with asterisks. Vertical bars designate significant differences between treatment groups. Wilcoxon matched pair test was used to assess statistical significance (*P<0.05, **P<0.01).

Stimulation of DC with H37Rv ManLAM significantly (P<0.05) increased their expression of CD80, CD86, and MHC II as compared with levels observed for unstimulated cells (Figure 2, P = 0.001, 0.001, and 0.016, respectively)). Unlike ManLAM, H37Rv PIMs alone did not affect surface expression of DC maturation markers during 48 h culture.

DCs from the same donor were also exposed to LPS or combination of LPS/PIM, LPS/ManLAM or LPS/PIM + ManLAM. Exposure to LPS resulted in maturation of DCs as manifested by increased expression of the activation markers CD80, CD86 and MHC II molecules (Figure 2 and Figure S1). ManLAM, when used together with LPS, potentiated the LPS-induced expression of CD80 (Figure 2A and Figure S1), but not the expression of CD86 (Figure 2B), or MHC II (Figure 2C). H37Rv-derived PIMs present in the culture medium reduced LPS-driven upregulation of MHC II but did not modulate LPS-induced expression of co-stimulatory molecules CD80 and CD86 on DCs (Figure 2 and Figure S1).

### Effect of *Mtb* ManLAM and PIM on DC Cytokine Production

In order to assess the impact of *Mtb* glycolipids on DC functional phenotype, TNF, IL-6, IL-12p40 and IL-10 were quantified by ELISA in supernatants from DCs cultured with ManLAM, PIMs and/or LPS. In preliminary experiments the DCs were exposed to LPS and *Mtb* H37Rv glycolipids for different time periods (not shown). Maximum release of TNF was noted after 8 h for LPS and 12 h for ManLAM stimulation. All subsequent cytokine secretion experiments were conducted with 12 h stimulation of DCs. Additionally, different glycolipids concentrations were tested in titration experiments where concentrations of 10 µg/ml for ManLAM and 5 µg/ml for PIMs were found optimal and used in all further DC experiments.

**Figure 3:**
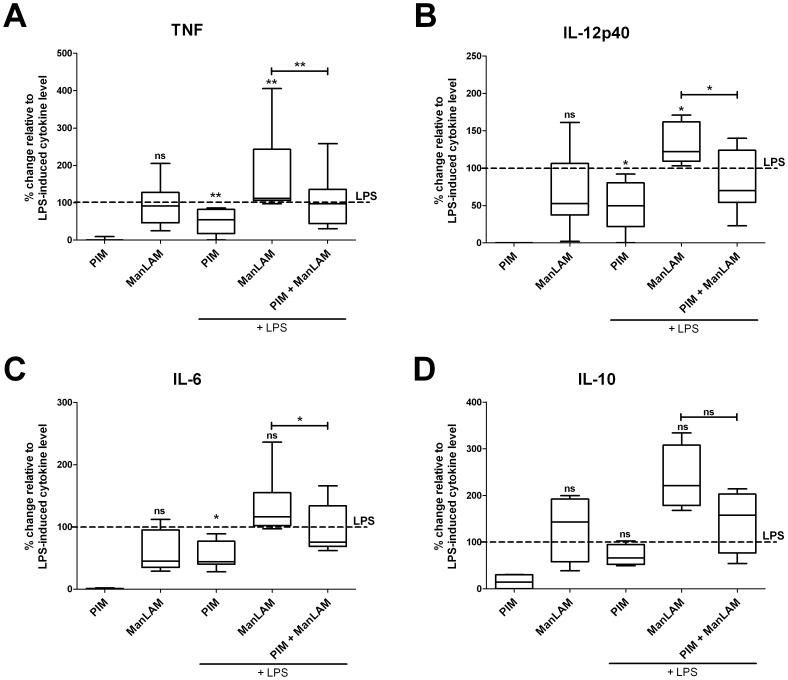
Cytokine production by DCs exposed to LPS and/or ManLAM and PIM from *Mtb* H37Rv. After 12 h exposure to ManLAM and/or PIM (at the concentration of 10 µg/ml and 5 µg/ml, respectively), TNF, IL-12p40, IL-6 and IL-10 in DC culture supernatants were assayed by ELISA. The level of cytokines released upon exposure to LPS (100 ng/ml) is shown as 100% of activation (dashed line). Background levels (not-treated cells) are 0%. The median percentage change in cytokine production is shown as a line. The box defines the 75th and 25th percentiles and the whiskers define the maximum and minimum values of 3–9 donors/group. Groups significantly different from LPS-treated control are labeled with asterisks. Vertical bars designate significant differences between treatment groups. Wilcoxon matched pair test was used to assess statistical significance (*P<0.05, **P<0.01).

H37Rv ManLAM stimulated DC production of all pro-inflammatory cytokines assayed (P<0.05). TNF was produced in quantities similar to those induced by LPS while IL-6 and IL-12p40 secretion reached 60–70% of LPS-induced levels (Figure 3A). When ManLAM was added to DCs together with LPS, it stimulated an increase of cytokine outputs over that induced by LPS alone (120–150%). *M. bovis* Pasteur 38152 ManLAM displayed a low but similar inductive activity as H37Rv ManLAM in terms of IL-6 and IL-12p40 secretion (Figure S2).

Unlike ManLAM, PIMs from H37Rv (Figure 3) and *M. bovis* (not shown) did not stimulate any pro-inflammatory cytokine production from DCs. Importantly however, PIMs from both H37Rv (Figure 3) and *M. bovis* Pasteur 38152 (not shown) inhibited by 40–50% LPS-induced secretion of TNF, IL-6 as well as IL-12p40. H37Rv PIMs also abrogated ManLAM-elicited augmentation of cytokine production in LPS-treated DCs (Figure 3). Inhibition of TNF and IL-12p40 production in DCs by H37Rv PIMs was dose-dependent in the range of 1–10 µg/ml (not shown).

The quantification of IL-10 revealed a similar pattern of cytokine release, where exposure of DCs to ManLAM triggered effects similar to those observed for LPS. However, inhibition of LPS-induced IL-10 production by PIMs did not reach statistical significance (Figure 3D).

### ManLAM-mediated Cytokine Induction does not Depend on Contamination with LPS or Lipopeptides

**Figure 4:**
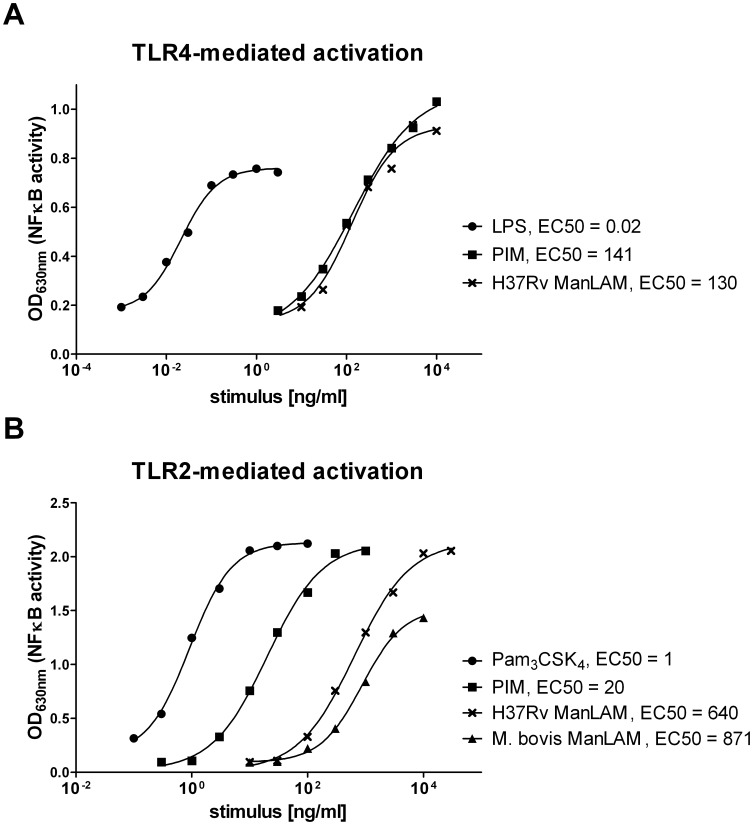
ManLAM- and PIM-mediated activation of TLR4 and TLR2 reporter cells. HEK-Blue™-hTLR4 (A) and HEK-Blue™-hTLR2 (B) cells were stimulated overnight with reciprocal dilutions of LPS (A), Pam_3_CSK_4_ (B), ManLAM from *M. bovis* (B) or H37Rv (A and B), or PIM (A and B). TLR4- and TLR2-mediated NF-κB activation was monitored by detection of NF-κB-controlled alkaline phosphatase secreted to the culture medium. EC50: half maximal effective concentration.

In order to exclude the possibility that the cytokine-inductive effect of ManLAM in DCs was due to contaminating LPS (endotoxin) or lipopeptides we investigated the effect of ManLAM on HEK-Blue™-hTLR4 and HEK-Blue™-hTLR2 cells, respectively. In TLR4 reporter cells the effect of ManLAM on NF-κB activation was negligible with an EC_50_ value of 130 ng/ml, which was over 3 orders of magnitude higher than that observed for LPS (EC_50_ of 0.02 ng/ml; Figure 4A). Similar low EC_50_ value of 141 ng/ml was noted for PIM in TLR4 reporter cells, indicating lack of LPS contamination (Figure 4A). In TLR2 reporter cells we compared the activating effect of Pam_3_CSK_4_, a classical lipopeptide agonist of TLR2, with those of ManLAM and PIMs. We confirmed that Pam_3_CSK_4_ was a strong NF-κB activator through TLR2 with an EC_50_ of 1 ng/ml (Figure 4B). By contrast, both H37Rv and *M. bovis* ManLAM showed very weak TLR2-mediated NF-κB activation (EC_50_ values of 640 ng/ml and 871 ng/ml, respectively) indicating no contamination with lipopeptides. As expected, PIMs was a much stronger TLR2 agonist than ManLAM with an EC_50_ of 20 ng/ml (Figure 4B). Similar data on ManLAM and PIMs binding to TLR2 were earlier published by others [42].

**Figure 5:**
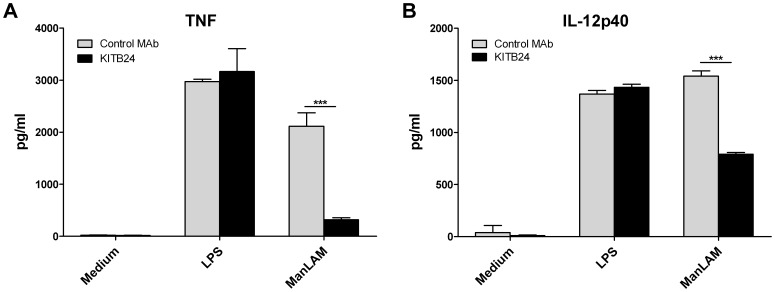
Inhibition of ManLAM-induced production of pro-inflammatory cytokines in DCs by ManLAM-specific MAb. ManLAM from *Mtb* H37Rv and LPS were pre-incubated for 1 h with ManLAM-specific MAb (KITB24) or isotype-matched control MAb. Next, MAb pre-incubated glycolipids (ManLAM and LPS at the concentration of 10 µg/ml and 100 ng/ml, respectively) were added to immature DCs and 12 h production of TNF (A) and IL-12p40 (B) was estimated in supernatants by ELISA. Experiment was carried out in triplicates, values are mean ± SD. Two-tailed, unpaired *t*-test was used to assess the statistical significance (***P<0.001).

**Figure 6:**
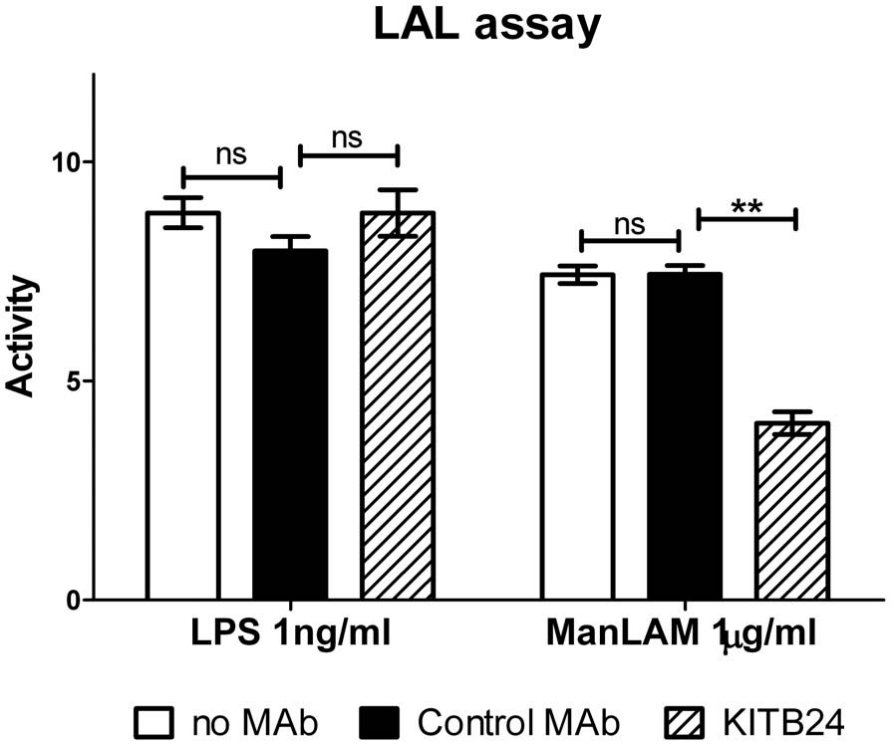
Limulus amebocyte lysate (LAL) assay of ManLAM. H37Rv ManLAM (1 µg/ml) and LPS (1 ng/ml) were tested for their activity in the LAL assay. Activity expressed as concentration of endotoxin in pg/ml was calculated according to the endotoxin standard included in the kit. In addition, in order to assess whether ManLAM associated activity in the LAL assay could result from LPS contamination, both lipoglycans were incubated for 1 h prior to LAL assay with ManLAM-specific MAb (KITB24) or isotype-matched control MAb. Experiment was carried out in triplicates, mean values ± SD are shown. *t*-test was used to assess the statistical significance of differences between indicated pairs (**P<0.01).

To further confirm that cytokine induction in DCs by ManLAM was not mediated by contaminating LPS, we examined the impact of the ManLAM-specific MAb KITB24, directed against the arabinomannan moiety on the H37Rv ManLAM-induced production of TNF and IL-12p40. Pre-incubation of our in-house H37Rv ManLAM with sub-molar amounts of MAb KITB24, but not with an irrelevant isotypic control MAb, abolished the ManLAM-induced production of TNF from DCs and reduced IL-12p40 secretion by 50%, but did not affect the LPS-induced production of the respective cytokines (Figure 5). Next, we examined the effect of the ManLAM-specific MAb on the “endotoxin-like” activity of ManLAM in the LAL assay. It is well known that both LPS and ManLAM are positive in this test [43] therefore we tested if activity detected in the LAL test could be blocked by the ManLAM-specific MAb KITB24. We found that while KITB24 MAb, even at a very low molar input ratio to ManLAM considerably (60%) reduced the ManLAM-associated activity it had no effect on the endotoxic activity of LPS in the LAL assay (Figure 6). Different concentrations of LPS and ManLAM used in this test were dictated by the sensitivity limits of the test and were chosen to fit in the linear phase of the standard curve.

## Discussion

Infection of MΦ and DCs with live mycobacteria is generally associated with induction of a strong pro-inflammatory phenotype with production of TNF, IL-12 and IL-6 that in turn is accompanied by the regulatory response, including secretion of IL-10. Thus, infection of human [44], [45], [46], [47] and mouse [48], [49] MΦ as well as human [44], [50], [51] and mouse [48], [52] DCs *in vitro* stimulates TNF and IL-12 transcription and secretion as well as maturation of DCs, as shown for *Mtb* [7], [44], [48], [52], *M. bovis* [53], BCG [44], [45], [50] and *M. avium* infections [51]. Elevated levels of IL-10 were observed in lungs and sera of patients with active TB and hypothesized to be responsible for impaired clearance of the pathogen at the early stages of *Mtb* infection [54]. Importantly, the maturation/activation state of DCs is critical to their responsiveness to infection [45].

The magnitude of the cytokine response in infected MΦ is highly mycobacterial strain-dependent. Thus, human alveolar MΦ infected with *Mtb* H37Rv or *M. bovis* produce more TNF than those infected with attenuated *Mtb* H37Ra or BCG [46]. Human MΦ [47] and mouse MΦ [45] infected with virulent clinical *Mtb* isolates in turn produce more TNF than those infected with *Mtb* H37Rv. We have found significantly increased production of TNF, IL-12p40 and IL-6 in co-cultures of human derived DCs with MΦ infected with two clinical *Mtb* strains, but not BCG, Copenhagen strain [55]. The reason for these strain-related differences is most likely multi-factorial, but could at least in part be dependent on the different nature of cell wall-associated molecules produced by the divergent mycobacterial strains.

Here, we report that highly purified ManLAM derived from the *Mtb* H37Rv strain triggers maturation of human DCs and production of pro-inflammatory cytokines, while ManLAM from *M. bovis* induces lower levels of pro-inflammatory cytokines. This difference in pro-inflammatory activity by ManLAM from the two strains could be explained by subtle but important differences in the structure of the respective ManLAMs [29]. Thus *Mtb* H37Rv and *M. bovis* BCG were reported to be heterogeneous with respect to arabinan and mannan domains and to differ in abundance of acyl-isoforms [56] which may well result in different biological activities.

### ManLAM from *Mtb* H37Rv Drives Maturation/Activation of Human DCs

Here, we demonstrate that H37Rv ManLAM is a potent activator of human DCs, inducing DC maturation of almost the same magnitude as LPS. This is in agreement with the findings of Dulphy *et al*. [57] but in contrast to the findings of Geijtenbeek and colleagues [25]. In the study by Dulphy *et al*. [57] DCs were activated with ManLAM from H37Rv, and were displaying a dose-dependent (from 0 to 10 µg ManLAM/ml) maturation phenotype, in terms of CD83 and CD86 expression. However, when compared to DCs stimulated with 10 ng/ml of LPS, the ManLAM-stimulated DCs showed an intermediate and delayed maturation phenotype. In the study by Geijtenbeek *et al.* [25] *Mtb* ManLAM did not induce DC maturation and, in contrast to our study, inhibited LPS-induced activation. The reason for this discrepancy is not clear but it could be hypothesized that strain-associated differences in the relative abundance of molecular forms of ManLAM or/and degree of purity could be responsible for the reported divergent effects on DC maturation (see below).

### ManLAM Induces a Pro-inflammatory Cytokine Pattern in Human DCs

In this study the H37Rv ManLAM induced a strong pro-inflammatory cytokine response in human DCs, manifested by TNF, IL-6, and IL-12 release, similar to that induced by LPS. A similar pattern was observed for IL-10. This was hardly surprising given that IL-10 is secreted in response to TLR ligation as well as high levels of TNF and IL-6 [58] in a negative feedback manner. Since high levels of IL-10 were observed in patients suffering from active TB and thought to be in part responsible for the impaired immunological balance seen in the course of *Mtb* infection [54] the elevated production of IL-10 observed here could add to the understanding of the *Mtb* pathology. The capacity of ManLAM to induce IL-10 has also been studied earlier; ManLAM was found to induce IL-10 from DCs [59] whereas in another study ManLAM was reported to be unable to trigger IL-10 release from human blood monocytes [60].

The induction of TNF was reminiscent of that observed in MΦ exposed to H37Rv ManLAM [35], [36], [39]. To our knowledge only one study has examined TNF production of DCs stimulated by ManLAM [40]. In that study two types of ManLAM obtained from BCG were reported to have different stimulatory capacity. One was cell wall-associated, “parietal” ManLAM which induced TNF and IL-8 production while the other was cell membrane-associated, “cellular” ManLAM which did not stimulate TNF or IL-8 [40]. The “cellular” ManLAM type also was reported to inhibit IL-12 secretion by human DCs [26]. The “parietal” type has unique fatty acids and was reported to be Man-capped to a larger extent than the “cellular” type [40]. Considering its TNF stimulatory effect, the H37Rv ManLAM investigated in the present study resembles the “parietal” ManLAM of Nigou *et al*.

Some of the discordant opinions about the effect of ManLAM on the induction of an inflammatory response by MΦ or DCs [9], stem from an early paper in the field [37], where ManLAM derived from the “virulent” laboratory Erdman strain was reported to produce a low pro-inflammatory response in terms of TNF induction in mouse MΦ compared to ManLAM from the “avirulent” H37Ra laboratory strain. Erdman ManLAM has since then been reported to induce significantly lower amounts of TNF not only compared to ManLAM from H37Ra [30], [31], [32] but also compared to ManLAM from the “virulent” H37Rv laboratory strain [39]. ManLAM from *Mtb* Erdman also failed to induce IL-12p40 in mouse MΦ, while AraLAM did [61]. Erdman ManLAM reportedly needs IFN-γ activation to stimulate TNF to the same extent as H37Ra [38], however in other studies bone marrow derived cells were shown to be unresponsive to Erdman ManLAM despite IFN-γ activation [62]. Thus the Erdman strain and its corresponding ManLAM seems to differ in terms of pro-inflammatory effect compared to other “virulent” laboratory strains such as H37Rv and virulent clinical isolates which are strong inducers of pro-inflammatory cytokines.

### H37Rv ManLAM Enhances LPS-mediated Stimulation of Pro-inflammatory Cytokines

As expected, LPS triggered production of all three pro-inflammatory cytokines TNF, IL-6, and IL-12, and H37Rv ManLAM together with LPS stimulated a moderate but significant increase of cytokine production over that induced by LPS alone. The finding that H37Rv ManLAM provided additional stimuli to the LPS-induced cytokine secretion resulting in the augmented cytokine output suggests that different receptors on human DCs may be involved in the ManLAM- as compared to LPS-driven cytokine responses.

Similar observations for IL-12p35, IL-12p40, IL-6 and IL-10 were made by Gringhuis *et al*. The production of cytokines was increased when cells were treated with LPS together with ManLAM as compared to cells exposed to LPS alone [63]. Strikingly, this is in contrast with the earlier results of Geijtenbeek *et al*. [25]; in their study neither LPS nor ManLAM, alone or in combination, induced significant amounts of IL-12p70. In the study by Geijtenbeek and colleagues the *Mtb* strain source, batch and purity of the ManLAM preparation obtained from TBVTRM is unclear.

### Comparison of In-house H37Rv and TBVTRM ManLAM Preparations

Differences in purification procedures of ManLAM may be another reason for the divergent results discussed above. TBVTRM ManLAM has for a long time been regarded as the gold standard and has been used by many investigators [63]. Therefore we undertook an analytical comparison of our in-house ManLAM and the TBVTRM ManLAM preparations.

Of the two TBVTRM ManLAM lots investigated here both contained PIMs and also some unidentified high molecular materials in addition to ManLAM. While our in-house H37Rv ManLAM preparation upon sugar analysis only showed trace amounts of glucose the TBVTRM lot tested showed a very high content of glucose suggesting contamination with mycobacterial glucans. Moreover of the 1 µg analyzed, our in-house ManLAM preparation contained 0.73 µg of expected sugars, while the TBVTRM lot tested only contained 0.55 µg.

Also when passing the TBVTRM ManLAM preparations through a HiTrap Phenyl-Sepharose column, we repeatedly found that about 50% of the material went through the column unretained. This unretained fraction was most likely arabinomannan (AM), devoid of most or completely lacking the lipid (acyl) part of ManLAM. This was supported by our finding that said fraction did not coat ELISA wells but was shown to maintain AM reactive epitopes in sandwich ELISA (using MAb KITB24 as capture antibody and biotinylated MAb KITB29 as detecting antibody).

Altogether these findings of contaminating material (glucans, PIMs) and potential lack of acylation in the TBVTRM ManLAM preparation have potential implications for the biological responses reported in the field, and may reconcile some differences seen where TBVTRM ManLAM preparations have been used. For example mycobacterial glucans have been reported to be ligands for DC-SIGN [64] and to block CD1 expression and suppress IL-12 production in monocyte-derived DCs [65]. The degree of acylation of mycobacterial glycolipids is important for their biological effects, e.g. the regulation of proinflammatory cytokines [22], [66], [67]. Thus, the presence of non-acylated or only partially acylated AM in a ManLAM preparation might exert a competitory/inhibitory effect on ManLAM. Further, one could hypothesize that high levels of contaminating PIMs may compete with or inhibit the cellular effects of a particular ManLAM lot.

### PIM Antagonizes LPS-induced Cytokine Production

Importantly, we here provide evidence that purified PIMs isolated from the same *Mtb* H37Rv cell wall preparation are potent inhibitors of the LPS-driven activation of human DCs. In addition, PIMs also abrogated the ManLAM-elicited augmentation of pro-inflammatory cytokine production in LPS-treated DCs.

Unlike ManLAM, PIMs alone did not affect surface expression of DC maturation markers during 48 h culture. Neither was LPS-induced DC maturation modulated by PIMs. Thus, the inhibition of LPS-induced cytokine production does not seem to be caused by delayed maturation/activation of DCs but is rather due to a down-regulation of either transcription and/or secretion of those cytokines. PIMs from *M. bovis* showed inhibitory activity similar to that of PIMs from H37Rv. This is consistent with the fact that both differently acylated isoforms, PIM_2_ and PIM_6_, are present in both H37Rv and *M. bovis* BCG [68].

### PIM as an Anti-inflammatory Agent

Different forms of LAM, LM and PIMs are all prevalent components of the mycobacterial cell wall [9]. PIMs and LM are direct precursors of LAM; PIM_2_ gives rise to the highly mannosylated LM molecule, which is further extended by the arabinan domain to form LAM. They are non-covalently attached to the plasma membrane through their phosphatidyl-*myo*-inositol anchor, and extend to the exterior of the cell wall [9]. PIM_2_ and PIM_6_ are the two most abundant classes of PIMs found in *Mtb* and BCG [23]. In earlier studies non-fractionated PIMs have been reported to stimulate TNF [20], [37], [69] through TLR2 signaling [21], [22]. According to these authors this effect depended on the presence of the lipid part of the molecule as de-acylation abrogated the TNF stimulation.

In a recent report PIM_2_ and PIM_6_ from BCG were reported to be anti-inflammatory, inhibiting LPS-induced TNF, IL-12, IL-6, and also IL-10 in mouse MΦ through a TLR2-independent mechanism [23]. The inhibitory effect depended on the acylation degree; 2-acylated and 3-acylated PIMs were inhibitory, 4-acylated were less inhibitory, and 1-acylated were non inhibitory. Our results corroborate observations of Doz and colleagues on a prominent anti-inflammatory action of PIMs [23] and extend these observations from murine MΦ to human DCs.

In conclusion, we here report that two major families of glycolipids of the mycobacterial H37Rv cell wall, ManLAM and PIMs, show profound but divergent effects on functional polarization of human DCs with regard to their maturation and pro-inflammatory cytokine responses. Highly purified H37Rv ManLAM induces a vigorous pro-inflammatory response while PIM is strongly anti-inflammatory. This implies that during *Mtb* infection *in vivo* ManLAM could contribute to a stimulation of a Th1 immune response whereas PIMs would rather down regulate the protective Th1 responses, competing with ManLAM and other pro-inflammatory types of *Mtb*-associated molecules. It is conceivable, that different LAM/PIM ratios in the cell wall of different bacterial strains (clones) and during distinct stages of *Mtb* infection may be a crucial factor in determining the differential stimulation or inhibition of the immune system and thereby be decisive for the emergence and outcome of the disease.

## Materials and Methods

### Cells

Human monocyte-derived DCs were prepared from peripheral blood mononuclear cells (PBMC) isolated from buffy coats obtained from healthy blood donors (Karolinska Hospital Blood Center, Stockholm). Cells were centrifuged on a density gradient (Lymphoprep, Axis-Shield, Norway) and CD14+ cells (monocytes) were isolated by positive selection using anti-CD14-coated magnetic microbeads (Miltenyi). The CD14+ cell population was more than 98% pure as assessed by flow cytometry (anti-CD14 APC-conjugated MAb, eBioscience). Monocytes were cultured for 5 days in the RPMI 1640 medium with 2 mM L-glutamine, 10% of FCS, and penicillin (50 U/ml) and streptomycin (50 µg/ml) (all from Gibco). Growth medium was supplemented with 75 ng/ml rhGM-CSF and 100 ng/ml rhIL-4 (both Peprotech). At days 2 and 5 a half of growth medium was replaced by fresh medium and new cytokines were added. For stimulation assays, cells at day 5 were replated from 6-well to the 96-well plate at 1.8×10^5^ cells/well.

### MAbs

MAb KITB24 recognizing arabinomannan and the MAb KITB51 recognizing PIM were produced in our laboratory.

### Reference LAM Preparations

ManLAM preparations produced at Colorado State University under the TBVTRM Contract were obtained from CSU (lot 08.Rv.1.24.ke1) and from the Biodefense and Emerging Infections Research Resources Repository (BEI Resources), USA (lot 09.Rv.2.9.8.ks), both prepared from H37Rv.

### Bacteria and Purification of LAM

The bacteria from *Mtb* strain H37Rv and from *M. bovis* (*M. bovis* strain 38152#, 1997, Institut Pasteur, Paris/France) were cultured on Löwenstein Jensen medium for 3–4 weeks and then propagated in Middlebrook 7H9 medium for 3 weeks, whereafter aliquots of bacilli were suspended in Middlebrook/10% glycerol and frozen at −70°C. Large scale cultures were grown similarly, cells were killed by heat-inactivation, frozen and thawed, several times, and subjected to gentle sonication whereafter crude cell wall preparations were freeze-dried. ManLAM and PIMs were purified using our earlier protocol [70] with some modifications. Briefly, dry cell walls of mycobacteria were rehydrated in PBS, sonicated and extracted in 40% hot phenol for 1 h at 70°C. Next, the dialyzed water phase was submitted to affinity chromatography on a Concanavalin A-Sepharose column. Bound material was eluted from the column and after buffer change subjected to hydrophobic interaction chromatography on a Phenyl-Sepharose column (Amersham, Sweden). Eluted glycolipid aggregates were then dissociated with deoxycholate (0.5%) at room temperature for 24 h followed by gel filtration on Sephacryl S-100 (Amersham, Sweden) in the presence of 0.25% sodium deoxycholate. Fractions containing ManLAM and PIMs were identified by Western blotting using MAbs specific to LAM and PIMs. Respective fractions were pooled and dialyzed under running water for 4 days to remove the detergent. The carbohydrate contents of glycolipids were determined by the phenol-sulfuric acid method [71]. SDS-PAGE of purified ManLAM and PIMs with periodate-silver staining was conducted using Phast System (Pharmacia Amersham, Sweden).

### Purification of PIM

We found that the phenol phase generated after extraction of mycobacterial cell walls was another rich source of PIMs. In order to purify PIMs from that phase we developed a novel method; the phenol phase was washed 3 times with PBS to remove residual LAM, LM and arabinomannan contaminants. The cleaned phenol phase was then mixed with an equal volume of 2% SDS in PBS and stirred overnight at room temperature. After phase separation by centrifugation the material in the water phase was precipitated with 9 vol of ice-cold ethanol. The precipitate was collected by centrifugation, solubilized with starting buffer consisting of 10 mM Tris-HCl, 0.5 M NaCl, 2 mM EDTA and 0.25% sodium deoxycholate pH 8.0 and chromatographed on S-100 equilibrated with the same buffer. The PIMs fractions emerged as a single peak free from any contaminants as ascertained by Western blotting and TLC. The fractions were then pooled and dialyzed against running water for 4 days or until no residual detergent was left.

### Characterization of Glycolipids

The ManLAM content was monitored at all purification steps by ELISA using MAb KITB24. The identity of the LAM preparation was confirmed by comparison with TBVTRM LAM (lot 08.Rv.1.24.ke1) in SDS-PAGE and Western blots, using anti-LAM MAb KITB24. The carbohydrate composition of ManLAM was analyzed by capillary electrophoresis with laser-induced fluorescence detection (CE-LIF) of 8-aminopyrene-1,3,6-trisulfonate (APTS) derivatives of sugars released by mild (0.1 N HCl, 30 min, 110°C) or total (2 M TFA, 2 h, 110°C) acid hydrolysis (courtesy of Dr. J. Nigou). PIM in low-molecular-weight (LMW) glycolipid fraction of H37Rv cell wall was detected in Western blots or immuno-dots and quantified in ELISA using MAb KITB51. The chemical identity of PIMs in the LMW glycolipid fraction was further confirmed by MALDI-TOF mass spectroscopy (courtesy of Drs. M. Gilleron and J. Nigou).

Further characterization of reference TBVTRM ManLAM (lot 08.Rv.1.24.ke1) was performed by hydrophobic interaction chromatography. Typically, 0.5 mg of TBVTRM LAM solubilized in 1 ml of 100 mM phosphate/0.8 M ammonium sulfate buffer pH 7.4 was slowly passed through 1 ml of a pre-packed HiTrap phenyl Sepharose column (Amersham). The flow through fraction constituting almost 50% of the starting material was saved and concentrated by rotary evaporation. Bound LAM was then eluted by 25% n-propanol, dialysed against PBS buffer and concentrated. Both fractions were then assessed for reactivity against anti-LAM MAb KITB24 in a sandwich ELISA. The flow through fraction material was not able to coat polystyrene microplates - most likely due to the lack of lipid moieties also as it evident by its inability to bind to the phenyl sepharose column. In the sandwich ELISA µg/ml of MAb KITB24 was used for coating and after blocking with 0.5% casein in PBS, different dilutions of the flow through fraction and the propanol eluted material (ranging from 200–50 ng/ml based on carbohydrate content) were examined. After washings, plates were developed by biotinylated anti LAM IgG MAb KITB29 followed by horseradish peroxidase conjugated Extravidin conjugate. The plates were then developed using TMB substrate and the absorbance was measured at 450 nm.

### Stimulation of DCs

Immature monocyte-derived DCs were stimulated at day 6 after separation of monocytes from the buffy coats and culture with rhGM-CSF and rhIL-4. For stimulation, LPS from *E. coli*, strain O127:B8 (Sigma) at the concentration of 100 ng/ml, ManLAM at a concentration of 10 µg/ml and PIM at the concentration of 5 µg/ml were used. In preliminary experiments these concentrations proved to give optimum cytokine induction in DCs. ManLAM, PIM and LPS stock solutions were prepared in saline. Cells were cultured in the presence of stimulants for 12 h after which supernatants were collected and stored at −80°C for cytokine assays. Cells for flow cytometry analysis were harvested and stained with antibodies after 48 h exposure to glycolipids. In MAb inhibition experiments ManLAM and LPS were pre-incubated with ManLAM-specific MAb (KITB24; 100 µg/ml) for 30 min, next the stimulants were added to DCs for 12 h after which culture supernatants were harvested for cytokine assays.

### TLR2 and TLR4 Reporter Cells

HEK-Blue™-hTLR2 and HEK-Blue™-hTLR4 cells (InvivoGen) were cultured in DMEM supplemented with 10% heat inactivated FBS, 50 U/ml penicillin, 100 µg/ml streptomycin, 2 mM L-glutamine (all from Gibco), 100 µg/ml Normocin and in the presence of HEK-Blue™ Selection antibiotics (both from InvivoGen). For stimulation experiments selection antibiotics were withdrawn from the culture medium. Cells were stimulated overnight with LPS, Pam_3_CSK_4_ and mycobacterial glycolipids at the concentrations indicated on the graph. The next day 25 µl of cell culture supernatant was mixed with QUANTI-Blue™ detection medium and incubated for another 2–3 h until the color was developed. Absorbance at 620 nm was measured.

### Flow Cytometry

FITC-, APC-, PerCP-, or PE-labelled antibodies used for flow cytometry analysis were: anti-MHC class II, anti-CD80, anti-CD86 (all from BD Biosciences), and anti-CD14 (eBioscience) with the appropriate isotype controls. Analyses were performed on the FACSCalibur instrument with CellQuest software (BD Biosciences) and processed by FlowJo (Tree Star).

### Cytokine Assays

Cytokines in supernatants from cell cultures (TNF, IL-12p40, IL-10 and IL-6) were quantified by ELISA (all from BD Biosciences, OptEIA™ sets), according to the manufacturer’s protocols.

### Limulus Amebocyte Lysate Assay

Limulus Amebocyte Lysate chromogenic endpoint assay (LAL; Hycult Biotechnology) was used to assess ManLAM’s “endotoxin-like” activity according to the manufacturer’s protocol. LAM-specific MAb KITB24 was used to block the ManLAM’s “endotoxin-like” activity in the LAL test. An isotypic MAb directed against irrelevant epitopes was used as negative control. In these experiments ManLAM or LPS were exposed to the MAbs (at a concentration of 100 µg/ml) for 1 h at room temperature and next subjected to the LAL assay.

### Statistical Analysis

Wilcoxon matched pair test or unpaired two-tailed *t*-test were used to assess the statistical significance. Data was analyzed using the GraphPad Prism software. Values of P<0.05 indicated statistical significance.

## Supporting Information

Figure S1 **CD80 expression on the surface of DCs exposed to different stimuli.** Flow cytometry histograms of DCs from one representative blood donor are shown. Dashed lines represent isotypic control, shaded histograms show CD80 expression level on DCs treated with saline (neg control, A–C) or LPS (D–F). Bold unshaded histograms show cells treated with LPS (A), PIM (B), ManLAM (C), PIM and LPS (D), ManLAM and LPS (E), or PIM, ManLAM and LPS (F).
(TIF)

Figure S2 **Cytokine production by DCs exposed to ManLAMs from** ***Mtb*** **H37Rv or** ***M. bovis*** **.** After 12 h exposure to ManLAMs from *Mtb* H37Rv or *M. bovis* or LPS, TNF, IL-12p40, IL-6 and IL-10 in DC culture supernatants were assayed by ELISA. Levels of cytokines released to medium by DC from a single donor are shown (mean of three wells ± SD).
(TIF)
