# Supplementary figures and images for: Divergent Effects of Mycobacterial Cell Wall Glycolipids on Maturation and Function of Human Monocyte-Derived Dendritic Cells
Source: PLoS One. 2012 Aug 3;7(8):e42515. doi: 10.1371/journal.pone.0042515 (PMC3411746; doi:10.1371/journal.pone.0042515)

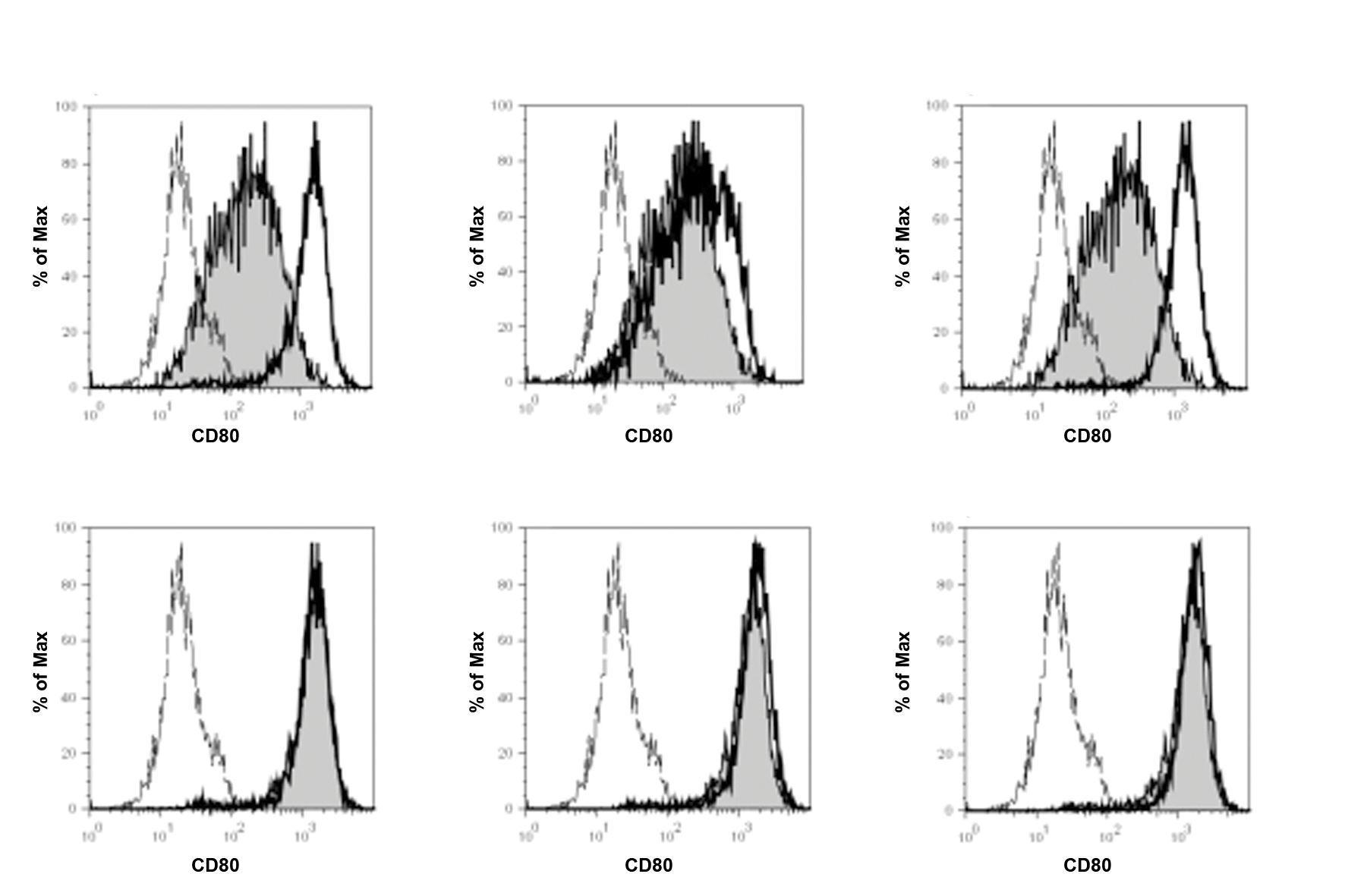

Supplement: Figure S1 — CD80 expression on the surface of DCs exposed to different stimuli. Flow cytometry histograms of DCs from one representative blood donor are shown. Dashed lines represent isotypic control, shaded histograms show CD80 expression level on DCs treated with saline (neg control, A–C) or LPS (D–F). Bold unshaded histograms show cells treated with LPS (A), PIM (B), ManLAM (C), PIM and LPS (D), ManLAM and LPS (E), or PIM, ManLAM and LPS (F). (TIF) [file pone.0042515.s001.tif]

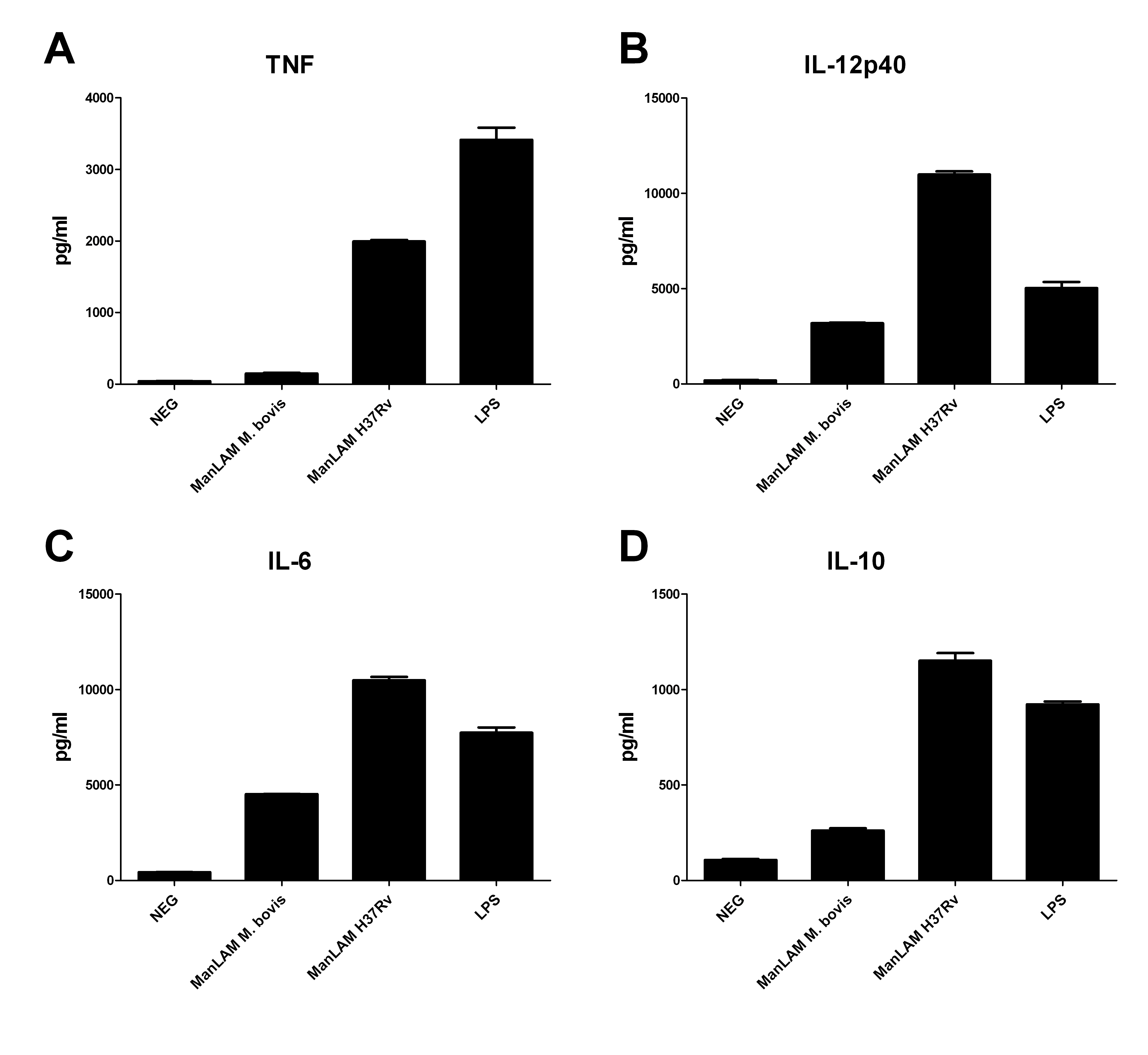

Supplement: Figure S2 — Cytokine production by DCs exposed to ManLAMs from Mtb H37Rv or M. bovis . After 12 h exposure to ManLAMs from Mtb H37Rv or M. bovis or LPS, TNF, IL-12p40, IL-6 and IL-10 in DC culture supernatants were assayed by ELISA. Levels of cytokines released to medium by DC from a single donor are shown (mean of three wells ± SD). (TIF) [file pone.0042515.s002.tif]
